# Comparison of posterior foraminotomy and anterior foraminotomy with fusion for treating spondylotic foraminal stenosis of the cervical spine: study protocol for a randomized controlled trial (ForaC)

**DOI:** 10.1186/1745-6215-15-437

**Published:** 2014-11-09

**Authors:** Anja Tschugg, Sabrina Neururer, Kai Michael Scheufler, Hanno Ulmer, Claudius Thomé, Aldemar Andres Hegewald

**Affiliations:** Department of Neurosurgery, Innsbruck Medical University, Innsbruck, Austria; Department of Medical Statistics, Informatics & Health Economics, Innsbruck Medical University, Innsbruck, Austria; Center for Spine Surgery, Hospital zum Heiligen Geist, Kempen, Germany; Department of Neurosurgery, University Medical Center Mannheim, Heidelberg University, Theodor-Kutzer-Ufer 1-3, 68167 Mannheim, Germany

**Keywords:** anterior cervical discectomy, anterior foraminotomy, cervical fusion, cervical spine, posterior foraminotomy, spinal surgery, spondylotic foraminal stenosis, randomized controlled trial

## Abstract

**Background:**

Cervical radiculopathy caused by spondylotic foraminal stenosis may require surgical treatment. Surgical options include anterior cervical foraminotomy and fusion or posterior cervical foraminotomy. Controversy remains regarding the preferable surgical approach. Pertinent clinical evidence is limited to low-quality observational reports. Therefore, treatment decisions are predominantly based on the individual surgeon’s preference and skill. The study objective is to evaluate the efficacy and safety of posterior foraminotomy in comparison to anterior foraminotomy with fusion for the treatment of spondylotic foraminal stenosis.

**Methods/design:**

This is a multicenter randomized, controlled, parallel group superiority trial. A total of 88 adult patients are allocated in a ratio of 1:1. Sample size and power calculations were performed to detect the minimal clinically important difference of 14 points, with an expected standard deviation of 20 in the primary outcome parameter, Neck Disability Index, with a power of 80%, based on an assumed maximal dropout rate of 20%. Secondary outcome parameters include the Core Outcome Measures Index, which investigates pain, back-specific function, work disability, social disability and patient satisfaction. Changes in physical and mental health are evaluated using the Short Form-12 (SF-12) questionnaire. Moreover, radiological and health economic outcomes are evaluated. Follow-up is performed 3, 6, 12, 24, 36, 48 and 60 months after surgery. Major inclusion criteria are cervical spondylotic foraminal stenosis causing radiculopathy of C5, C6 or C7 and requiring decompression of one or two neuroforaminae. Study data generation (study sites) and data storage, processing and statistical analysis (Department of Medical Statistics, Informatics and Health Economics) are clearly separated. Data will be analyzed according to the intention-to-treat principle.

**Discussion:**

The results of the ForaC study will provide surgical treatment recommendations for spondylotic foraminal stenosis and will contribute to the understanding of its short- and long-term clinical and radiological postoperative course. This will hopefully translate into improvements in surgical treatment and thus, clinical practice for spondylotic foraminal stenosis.

**Trial registration:**

Current Controlled Trials: ISRCTN82578069.

**Electronic supplementary material:**

The online version of this article (doi:10.1186/1745-6215-15-437) contains supplementary material, which is available to authorized users.

## Background

Cervical radiculopathy is a clinical diagnosis defined by the presence of sensory or motor deficits and complaints caused by mechanical compression of the corresponding cervical nerve root. Degenerative etiologies include disc herniation, spinal canal stenosis and spondylotic foraminal stenosis. Cervical radiculopathy remains a potentially disabling disease with a significant impact on the patient’s quality of life. Despite adequate conservative non-operative therapy, a large number of patients will require surgical treatment. Widely used options in this setting include anterior cervical discectomy and fusion, cervical arthroplasty, and posterior cervical foraminotomy. Moreover, a significant increase in the frequency of surgical treatment has been reported within the past decade. From 1999 to 2008, the annual number of cervical discectomies with subsequent fusion for degenerative disc diseases in the USA increased by 67% [1]. In a larger retrospective survey, however, spondylotic foraminal stenosis accounted for only approximately 20% of the surgical interventions in the degenerative cervical spine [2]. Of these, around two-thirds were due to unilateral foraminal stenosis, whereas one-third comprised decompression of two or more stenotic foramina, emphasizing the multifocal nature of the disease. Although posterior approaches to the cervical spine had been described almost a decade earlier than the anterior approaches [3, 4], anterior discectomy has become the gold standard for treating cervical degenerative disc diseases resulting in encroachment of the central spinal canal [5–7]. For lateral pathologies, however, there is still controversy regarding the most adequate surgical approach. Most pertinent literature in this matter consists of low-quality observational reports. Analyses of three comparative studies on the treatment of cervical disc herniation yield inconclusive results. One group of investigators observed an advantage in the anterior approach in terms of short-term neurological recovery [8]. In turn, other authors have reported significant benefits of the posterior approach regarding the average time to return to unrestricted full duty of military personnel [9]. A low-quality randomized controlled trial comparing the two surgical approaches in the treatment of acute cervical disc herniations did not show any significant difference in outcome [10]. To the best of our knowledge, prospective comparative data primarily investigating spondylotic foraminal stenosis is still lacking. Even non-comparative retrospective data on spondylotic foraminal stenosis is scarce. Studies on posterior foraminotomy reported good outcomes in 64% to 96% of patients and re-operation rates of around 4% to 7% [11–13]. The few studies exclusively investigating anterior cervical foraminotomy and fusion reported 83% to 91% good results and re-operation rates of 4% to 14% [14, 15]. Possible advantages of anterior cervical foraminotomy are direct access to remove anterior osteophytes and the option of decompressing the foramina bilaterally using a single surgical access. Moreover, the insertion of a cage provides an additional indirect decompressive effect of the foramina. This approach also offers the important option of sagittal correction in cases with segmental kyphosis. A potential disadvantage of anterior fusion might be the precipitation of adjacent segment disease. In reviewing 409 anterior cervical fusions, Hilibrand and colleagues [16] reported a relatively constant rate of symptomatic adjacent segment disease of 2.9% per year, with 25.6% of patients developing adjacent segment disease within 10 years of the operation and 7.5% of these patients requiring a re-operation. By contrast, single-level posterior foraminotomy for cervical radiculopathy displayed an annual 0.7% rate of adjacent segment disease cumulating to 6.7% at 10 years and a re-operation rate of 3.2% [17]. A potential advantage of posterior cervical foraminotomy is the maintenance of segmental motion in the majority of treated segments, reducing the likelihood of adjacent segment degeneration, as outlined. Two major concerns with posterior foraminotomies are: (1) persistent neck and shoulder pain secondary to muscle stripping performed during the conventional open approach and (2) same-level degeneration and kyphosis secondary to partial resection of the facet joint. The latter issue is the subject of some controversy. A frequently cited *in-vitro* study has shown that non-physiological segmental mobility of the cervical spine occurs when posterior foraminotomy involves resection of more than 50% of a facet joint [18]. However, other studies demonstrated that, even in cases of extensive facetectomy, a cervical motion segment will remain stable if all the anterior elements and one additional posterior element, such as the interspinous and supraspinous ligaments, are left intact [19, 20]. A clinical study, supporting these biomechanical results, reported favourable outcomes in more than 90% of the patients five years after posterior cervical foraminotomy including routine removal of 75% of the facet joint [21].

The study objective is to evaluate the efficacy and safety of posterior foraminotomy compared with anterior foraminotomy with fusion for the treatment of spondylotic foraminal stenosis. Both surgical approaches are well-established techniques in clinical practice and can be performed with comparable low risk. The surgical risk profiles of the approaches differ according to local anatomical features. Anterior surgery includes risks of injury of cervical viscera, nerves (laryngeal recurrent nerve, sympathetic chain) and vessels. Moreover, placement of an intervertebral fusion cage might potentially result in implant dislocation, pseudarthrosis and adjacent segment disease. Specific risks of the posterior approach are advancing degeneration of the affected level and progressive kyphotic deformity. Participation in the study does not result in specific benefits for the patient.

## Methods/design

### Study design

The ForaC study is a multicenter randomized, controlled, parallel group superiority trial with 88 adult patients allocated to the groups in a 1:1 ratio. The expected enrolment time is 2 years, and the conclusion of the study is estimated at 7 years. The primary study endpoint is the difference in Neck Disability Index between treatment groups at five years after intervention. As one of the secondary study endpoints, the Core Outcome Measures Index is applied to assess pain, back-specific function, work disability, social disability and patient satisfaction. Moreover, changes in physical and mental health are assessed by the Short Form-12 (SF-12) questionnaire, version 2. Adjacent level degeneration, segmental lordosis and overall cervical sagittal alignment are determined by flexion or extension X-rays and magnetic resonance imaging. Pre-operative American Society of Anesthesiologists grade staging might allow the identification of risk factors. Neurological status and the quality and quantity of current pain medication are documented. Operation time and time of hospitalization are documented. Quantitative sensory testing is performed to assess and quantify sensory nerve function non-invasively. We also evaluate the direct costs of hospital care and the indirect costs of follow-up treatment outside the hospital. Costs of surgery and hospitalization, including duration of inpatient treatment, cost of nursing, costs of medication and physiotherapy, are assessed after discharge from the hospital according to internal cost-estimate lists from the hospital operator. In addition, the indirect costs following discharge from hospital (for example, including physiotherapy, rehabilitation centres, pain medication, medical consulting) are documented on a routing sheet. Each study participant will receive such a routing sheet when leaving the hospital, with instructions for documentation. Postoperative care is not standardized in the study protocol and is to be performed according to the standard of care for spondylotic foraminal stenosis surgery at the participating sites. An external orthosis is not used in either group. Primary and secondary outcome parameters are outlined in more detail in Table 1. Ethics approval was attained at the local research ethics committee of the Medical University Innsbruck (UN4702). This study complies with the World Medical Association Declaration of Helsinki Ethical Principles for Medical Research Involving Human Subjects, 2008.

**Table 1:** **Primary and secondary outcome parameters**

Primary outcome parameter	Neck Disability Index at five years’ follow-up
Secondary outcome parameters	Core Outcome Measures Index
	Individual patient success at 12, 24, 36, 48 and 60 month defined as:
	● Improvement of at least 17 in the Neck Disability Index (100 points) compared with baseline (adjustable according to results from own minimal clinically important change results for Neck Disability Index).
	● Pain relief, as defined by ≥20 mm improvement on 100 mm visual analog for arm/shoulder pain
	● Global outcome (1 or 2 on five-category Likert scale)
	● No opiates or opiate derivatives because of neck or arm pain
	● Absence of symptomatic device failure and re-operations at the index level
	Pain relief, as defined by ≥20 mm improvement on 100 mm visual analog scale for neck pain and arm or shoulder pain
	Changes in physical and mental health defined as improvement of 15% in the overall score as captured by the Short Form-12 (SF-12) version 2 questionnaire (Brazier 2005)
	Modified Japanese Orthopedic Association score and Nurick score
	Adjacent level degeneration:
	● By evidence of instability, defined as sagittal plane translation >3.5 mm (20% of vertebral body anterior-posterior diameter) or sagittal plane rotation of >20° based on standing flexion or extension X-rays
	● By evidence of disc degeneration (Miyazaki grade ≥ IV) or osteochondrosis (Modic change type I) on magnetic resonance imaging
	● Radiographic classification (Walraevens 0 to 3)
	● By occurrence of operation because of adjacent level disease
	Quantitative sensory testing
	Segmental lordosis and overall cervical sagittal alignment
	Operative time
	Length of hospital stay
	Pain medication usage (including epidural injections and nerve block injections)
	Return to work
	Worker’s compensation
	Direct and indirect societal costs

### Study population

The ForaC study aims to include patients who qualify for decompression of ≤2 cervical neuroforamina by a posterior or an anterior approach because of spondylotic foraminal stenosis with radiculopathy. The target population consists of patients with symptomatic spondylotic foraminal stenosis without central canal stenosis who failed adequate conservative or interventional therapy administered for a minimum of six weeks. Radiculopathy is defined as pain, paralysis or paresthesia in a specific nerve root distribution at C5, C6 or C7. To recruit a rather homogeneous patient population, patients with less severe symptomatology (Neck Disability Index <30 points out of 100) are excluded. Additionally, a radiologically (computed tomography and magnetic resonance imaging) determined pathology at treatment level needs to correlate with the primary symptoms. To minimize risk factors for an unfavourable outcome, patients with significant comorbidities need to be excluded, as this may mask a difference in treatment efficacy. Inclusion and exclusion criteria are outlined in more detail in Table 2.

**Table 2:** **Inclusion and exclusion criteria**

Inclusion criteria	Age between 18 and 80 years
	Cervical spondylotic foraminal stenosis causing radiculopathy of C5, C6, or C7 and requiring decompression of ≤2 neuroforaminae
	Radiculopathy is defined as pain, paralysis or paresthesia in corresponding nerve root distribution areas of C5, C6, or C7, and must include at least arm or shoulder pain with minimum of 30 mm on a 100 mm visual analog scale
	Neck Disability Index score ≥30 out of 100
	Unresponsive to non-operative treatment for six weeks or presence of progressive symptoms or signs of nerve root compression in the face of conservative treatment
	Spondylotic foraminal stenosis (determined by magnetic resonance imaging and computed tomography) at treatment level correlating to primary symptoms
	Appropriate candidate for treatment using either of:
	● Anterior approach via ventral discectomy and fusion
	● Posterior approach via foraminotomy, as described by Frykholm
	Psychosocially, mentally, and physically able to fully comply with this protocol, including adhering to scheduled visits, treatment plan, completing forms, and other study procedures
	Personally signed and dated informed consent document prior to any study-related procedures, indicating that the patient has been informed of all pertinent aspects of the trial
Clinical exclusion criteria	Previous cervical spinal surgery at index level
	Lumbar or thoracic spinal disease to the extent that surgical consideration is probable or anticipated within 6 month after the cervical surgical treatment
	Upper extremity degenerative joint diseases (that is, shoulder) to the extent that:
	● Surgical consideration is likely or anticipated within 6 month after the cervical surgical treatment
	● Resulting pain is chronic (>3 month)
	Axial neck pain in the absence of other symptoms of radiculopathy justifying the need for surgical intervention
	Myelopathy
	Neoplasia as the source of symptoms
	Fixed or permanent neurological deficit unrelated to the cervical disc disease
	Disease or conditions that preclude accurate clinical evaluation (for example, neuromuscular disorders)
	Active or chronic infection, systemic or local
	Systemic disease, including HIV, AIDS, or hepatitis
	Active malignancy defined as a history of any invasive malignancy, except non-melanoma skin cancer, unless the patient has been treated with curative intent and there have been no clinical signs or symptoms of the malignancy for a minimum of 5 years
	Paget’s disease, osteomalacia, or any other metabolic bone disease
	Autoimmune disorder that impacts the musculoskeletal system (that is, lupus, rheumatoid arthritis, or ankylosing spondylitis)
	Acute episode or major mental illness (psychosis, major affective disorder or schizophrenia)
	Physical symptoms without a diagnosable medical condition to account for the symptoms, which might indicate symptoms of psychological rather than physical origin
	Recent or current history of substance abuse (drugs, alcohol, narcotics, recreational drugs)
	Anticipated long-term use of systemic steroid medications postoperatively
Radiological exclusion criteria	Symptomatic spondylotic foraminal stenosis, considered for surgical intervention, with contralateral asymptomatic spondylotic foraminal stenosis at the same level with equal or higher extent, as shown by computed tomography
	Cervical disc herniation or central canal stenosis causing radiculopathy or clinical myelopathy
	Myelopathy, as shown by magnetic resonance imaging
	Marked cervical instability on flexion or extension radiographs defined as:
	● Translation >3 mm or
	● Angulation >20°
	Kyphotic segmental angulation >11° at treatment or adjacent levels
	VARIA
	Patient is currently pursuing personal litigation related to spinal diseases
	Prisoner or ward of the state
	Patient has used another investigational drug or device within the 30 days prior to surgery

Informed consent is obtained from each participant.

### Timetable

The timetable and visit plan is outlined in Table 3.

**Table 3:** **Visit plan**

	Pre-operation	Intra-operation	Post-operation, 3 days	Discharge	3 months (±2 weeks)	6 months (±1 month)	12 months (±2 months)	24 months (±2 months)	36 and 48 months (±2 months)	60 months (±2 months)
Informed consent	×	-	-	-	-	-	-	-	-	-
Pre-operative history	×	-	-	-	-	-	-	-	-	-
Randomization	×	-	-	-	-	-	-	-	-	-
American Society of Anesthesiologists grade	×	-	-	-	-	-	-	-	-	-
Operative detail	-	×	-	-	-	-	-	-	-	-
Clinical evaluation: neurological status, pain medication consumption	×	-	×	-	×	×	×	×	×	×
Patient self-assessment: Neck Disability Index, Core Outcome Measures Index, Short Form-12 (SF-12) questionnaire	×	-	×	-	×	×	×	×	×	×
Quantitative sensory testing	×	-	-	-	-	×	×	-	-	-
Schedule of radiographic studies										
Neutral, lateral and anterior-posterior	×	×	-	-	×	×	×	×	-	-
Flexion or extension	×	-	-	-	×	×	×	×	-	×
Computed tomography	×	-	×	-	-	-	-	-	-	-
Magnetic resonance imaging	×	-	-	-	×	-	-	×	-	×
Economic data collection										
Hospitalization costs	-	-	-	×	-	-	-	-	-	-
Post-hospitalization costs	-	-	-	-	×	×	×	×	×	×

### Investigational groups

#### Posterior foraminotomy

A midline skin incision is made extending across the cervical motion segments of interest. The neck muscles are subperiosteally dissected from the bone to expose the lamina and a retractor system is applied. Once the facet joint complex is exposed, a Kerrison punch is used to remove some of the medial superior and inferior lamina to access the spinal canal. A microscope is used for improved illumination and visualization. A high-speed drill is then used to thin the medial facet, centred over the joint. The remaining bone overlying the nerve root is removed using angled curettes and small Kerrison instruments. The nerve hook is passed laterally out through the foramen to confirm adequate neural decompression [4, 21].

#### Anterior foraminotomy

A transverse skin incision of 3 to 4 cm is made over the cervical motion segments of interest. Access to the cervical column is prepared by sharp and blunt dissection, opening the superficial fascia at the medial border of the sternocleidomastoid. Under distraction of the target level, a discectomy is performed with full exposure of the posterior longitudinal ligament. Adjacent vertebral osteophytes are then resected with complete foraminotomy on the affected side, with use of the operating microscope. Direct visualization of the involved nerve root from the axillae up to 3 mm distally is obtained. The distraction is released, locking the intervertebral cage firmly into position. A ventral locking plate is used for additional stability, if deemed necessary [14].

#### Randomization

**Figure 1:**
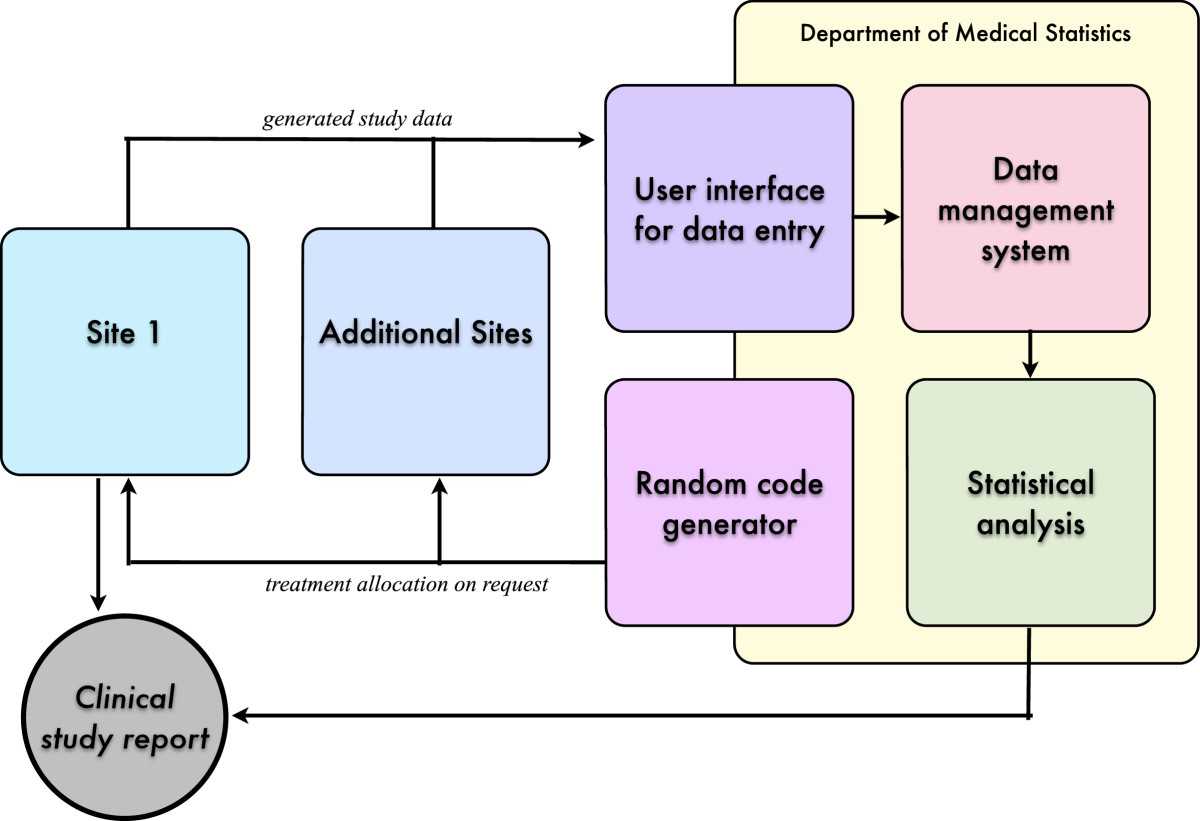
**Data management and randomization process.** Study data generation at the study sites is clearly separated from data storage, processing and statistical analysis.

The allocation ratio is 1:1. The randomization code will be generated independently from the clinical investigators according to a random permuted blocks method with varying block size. Randomization will be stratified according to study centres. Statistical Software Stata 10.0 module Ralloc version 3.5.2 (Statacorp College Station, TX, USA) will be used to generate the random code. An independent statistician at the Department of Medical Statistics, Informatics and Health Economics, Innsbruck Medical University will administer the randomization code (Figure 1).

#### Data management

Study data generation at the study sites is clearly separated from data storage, processing and statistical analysis in the Department of Medical Statistics, Informatics and Health Economics (Figure 1). This requires a validated database system programmed in a customized software package, which is provided by the Department of Medical Statistics, Informatics and Health Economics of the Medical University, Innsbruck. The system includes an audit trail facility and will be used to define the database structure, data entry, for handling data cleaning processes, and for final data storage. Data evaluation takes place by double entry of the data and manual and visual evaluation of plausibility. After entry of all collected data and clarification of all queries, the database will be closed at the completion of the study. This performance has to be documented.

#### Sample size and power calculation

The sample size calculation is based on the primary endpoint of the study, the group difference in the Neck Disability Index. A reduction in Neck Disability Index score of 14 to 17 points out of 100 is considered to be clinically meaningful [22, 23]. Thus, 14 points were used as the required difference between groups applicable for the power calculations. Moreover, a group standard deviation of 20 is assumed, based on results of comparable previous trials [24, 25]. A sample size calculation was performed based on a two-group *t* test (GraphPad StatMate Software, version 2.0); 35 patients per group are required to detect a 14-point difference with 80% power on a two-sided level of significance of 0.05. A loss to follow-up of less than 20% was considered adequate for final calculation of sample size. Accordingly, the proposed sample size amounts to 88 patients, that is, 44 patients in each group. In case of non-normality of the Neck Disability Index, this sample size is also sufficient to detect a difference of 14 points with a Mann-Whitney *U* test.

#### Statistical analysis

Endpoints will be analyzed as appropriate depending on data distribution with a two-sided 0.05 level of significance. Detailed descriptive statistics will be provided for the data collected and 95% confidence intervals will be calculated for all relevant estimates. Clinical follow-up data will be analyzed by analysis of covariance (ANCOVA) or generalized model alternatives for categorical or semiquantitative data. Changes within the treatment groups over time as well differences between groups will be assessed simultaneously. The primary analysis will follow the intention-to-treat principle. All randomized patients with a complete preliminary examination will be considered for inclusion into the intention-to-treat population. Further sensitivity analysis will be provided to evaluate robustness of the results with regard to unexpected circumstances (for example, impact of ‘cross-over’ patients who are not treated as randomized but are required to be analyzed as randomized (intention-to-treat principle) and centre effects. Secondary endpoints will be analyzed in an exploratory manner at a two-sided significance level of 5%. Safety and tolerability parameters will be analyzed descriptively. Frequencies will be compared by Chi-square or Fisher’s exact tests, as appropriate. Analysis of time-dependent probabilities of critical events will be performed using the Kaplan-Meier method. Furthermore, multivariate event analyses will be performed using Cox proportional hazard regression models. The last-observation-carried-forward approach will be employed, in order to perform an intention-to-treat analysis of the primary efficacy endpoint in consideration of all randomized patients. In addition, for the purpose of a supportive sensitivity analysis, multiple imputation procedures will be applied. Statistical analysis will be performed 12, 24, 36 and 60 months after completion of the last visit of the study population at the specified time points.

## Discussion

Owing to the high prevalence of spinal diseases and increasing numbers of spinal interventions, spine research has partially become an industry-driven field. These factors have facilitated large clinical trials on several frequent spinal diseases. However, less common spinal diseases not requiring special spinal implants and instruments have not been subjected to well-designed clinical trials. This phenomenon also applies to spondylotic foraminal stenosis, representing approximately 20% of degenerative cervical conditions requiring surgical intervention. The majority of clinical data on spondylotic foraminal stenosis are more than 10 years old. Beside the fact that there is a lack of comparative data, it is also important to acknowledge that clinical outcomes reported in these historic studies were primarily based on rather crude rating scales of the patient’s condition, and clinical follow-up was exclusively performed by the treating physicians. With current standards, patient-centred, well-validated outcome instruments are mandatory and there is an urgent need for comparative data with validated modern outcome instruments to develop evidence-based treatment recommendations. At this time, there are no evidence-based guidelines on the most appropriate surgical treatment strategy for cervical spondylotic foraminal stenosis. Treatment decisions are determined predominantly by the individual surgeon’s preference and skill. The results of this study will provide surgical treatment recommendations for spondylotic foraminal stenosis and contribute to the understanding of its short- and long-term clinical and radiological postoperative course. This will hopefully be translated into an improvement of surgical strategy and thus, clinical practice for spondylotic foraminal stenosis.

## Trial status

The trial started in June 2013 with two sites in Austria, the Department of Neurosurgery, Medical University Innsbruck and the Department of Neurosurgery, LKH Feldkirch. Two sites in Germany will join as soon as local ethical approval is granted: the Department of Neurosurgery, University Medical Center Mannheim, Heidelberg University and the Spine Center, Hospital zum Heiligen Geist Kempen. More sites might be recruited during the enrolment phase.

## Authors’ original submitted files for images

Below are the links to the authors’ original submitted files for images. Authors’ original file for figure 1

## Abbreviations

ANCOVA: analysis of covariance
SF-12: Short Form-12.
