# Supplementary figures and images for: Comparison of posterior foraminotomy and anterior foraminotomy with fusion for treating spondylotic foraminal stenosis of the cervical spine: study protocol for a randomized controlled trial (ForaC)
Source: Trials. 2014 Nov 9;15:437. doi: 10.1186/1745-6215-15-437 (PMC4289374; doi:10.1186/1745-6215-15-437)

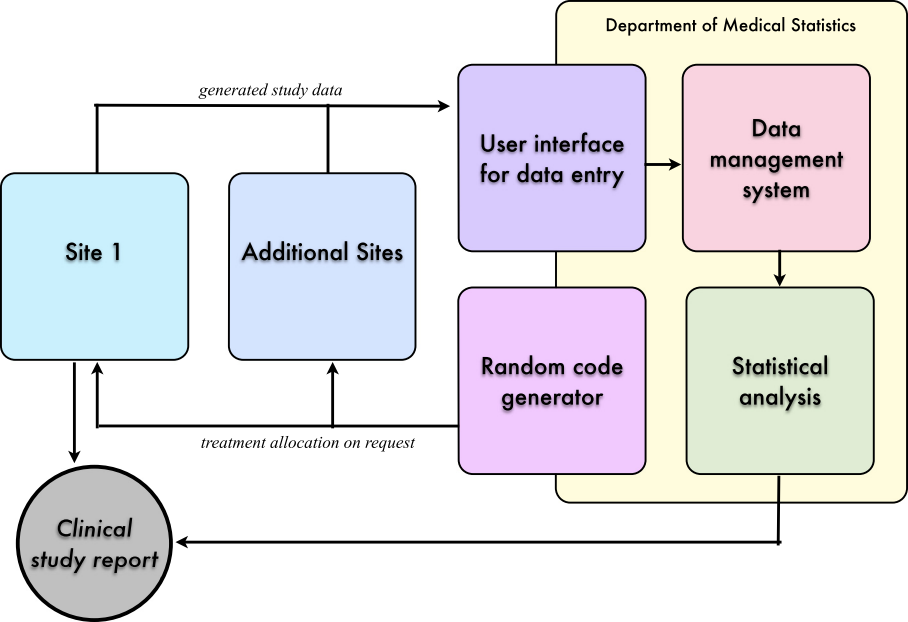

Supplement: Supplementary file 1 — Authors’ original file for figure 1 [file 13063_2013_2362_MOESM1_ESM.pdf]
